# Acknowledgement to the Reviewers of GMS Journal for Medical Education

**DOI:** 10.3205/zma001209

**Published:** 2019-02-15

**Authors:** Martin R. Fischer, Götz Fabry

**Affiliations:** 1 GMS Journal for Medical Education, Chief Editor, Erlangen, Germany; 2 Klinikum der Universität München, Institut für Didaktik und Ausbildungsforschung in der Medizin, München, Germany; 3 GMS Journal for Medical Education, Assistant Chief Editor, Erlangen, Germany; 4 Albert-Ludwig-Universität Freiburg, Medizinische Fakultät, Abteilung für Medizinische Psychologie und Soziologie, Freiburg, Germany

## Acknowledgement

We gratefully acknowledge the valuable contribution of the following peer reviewers, who supported our Journal in 2018. We are looking forward to continuing our collaboration!

## Reviewers in alphabetical order

- Abdullah Ahmed, Heidelberg
- Helmut Ahrens,Münster
- Obada al Halabi, Heidelberg
- Karam Alhalabi, Heidelberg
- Simone Alvarez, Heidelberg
- Stephanie Alvino, Bonn
- Ali Amr, Heidelberg
- Matthias Angstwurm, München
- Christina Armstrong, Heidelberg
- Timo Astfalk, Rostock
- Birgit Babitsch, Osnabrück
- Cadja Bachmann, Rostock
- Claas Baier, Hannover
- Anne Barzel, Hamburg
- Claudia Bausewein, München
- Pascal Berberat, München
- Martin Bergold, Oldenburg
- Florian Beuer, Berlin
- Stephan Birk, Berlin
- Monika Bobbert, Münster
- Martin Boeker, Freiburg i. Br.
- Patrick Boldt, Düsseldorf
- Raphael Bonvin, Fribourg (CH)
- Myrto Boukovala, München
- Barbara Braun, Heidelberg
- Jan Breckwoldt, Zürich (CH)
- Erich Brenner, Innsbruck (AT)
- Monika Brodmann Maeder, Bern (CH)
- Till Bugaj, Heidelberg
- Andreas Burger, Bochum
- Jamiu Busari, Maastricht (NL)
- Beatrice Buss, Bern (CH)
- Alexandra Castell Morley, Heidelberg
- Jean-Francois Chenot, Greifswald
- Jan de Lafollie, Gießen
- Ulrich Decking, Düsseldorf
- Antje Degel, Berlin
- Peter Dieckmann, Herlev (DK)
- Christoph F. Dietrich, Bad Mergentheim
- Claudius Diez, Halle
- Winand Dittrich, Essen
- Sabine Drossard, München
- Julia Eckel, Mannheim
- Jan P. Ehlers, Witten
- Maren Ehrhardt, Hamburg
- Rebecca Erschens, Tübingen
- Götz Fabry, Freiburg i. Br.
- Nabeel Farhan, Freiburg i. Br.
- Ricardo Felberbaum, Kempten
- Volkhard Fischer, Hannover
- Peter Frey, Bern (CH)
- Rainer Gaupp, Basel (CH)
- Karim Gawad, Bielefeld
- Kirsten Gehlhar, Oldenburg
- Franziska Geiser, Bonn
- Waltraud Georg, Zürich (CH)
- Susanne Gerhardt-Szép, Frankfurt/Main
- Marianne Giesler, Freiburg i. Br.
- Maryna Gornostayeva, Mainz
- Lorenz Grigull, Hannover
- Christian Gruber, Hannover
- Markus Gulich, Ulm
- Jennifer Guse, Hamburg
- Rainer Haak, Leipzig
- Petra Hahn, Freiburg i. Br.
- Antje Hammer, Bonn
- Judith Hammerschmidt, Bonn
- Roman Hari, Bern (CH)
- Cordula Harter, Heidelberg
- Anja Härtl, Augsburg
- Wolf Hautz, Bern (CH)
- Inga Hege, Augsburg
- Nicole Heitzmann, München
- Gunther Hempel, Leipzig
- Michael Hempel, Leipzig
- Tanja Hitzblech, Berlin
- Verena Hofbauer, Wien (AT)
- Matthias Hofer, Düsseldorf
- Anke Hollinderbäumer, Mainz
- Henrike Hölzer, Neuruppin
- Angelika Homberg, Heidelberg
- Marion Huber, Winterthur (CH)
- Markus Huber-Lang, Ulm
- Sören Huwendiek, Bern (CH)
- Paul Jansen, Kamen-Heeren
- Caroline Jung-Sievers, München
- Sylvia Kaap-Fröhlich, Zürich (CH)
- Marco Kachler, Berlin
- Martina Kadmon, Augsburg
- Hanna Kaduszkiewicz, Kiel
- Juliane Kämmer, Berlin
- Yassin Karay, Köln
- Kianush Karimian Jazi, Heidelberg
- Gudrun Karsten, Kiel
- Matthew Kerry, Zürich (CH)
- Jan Kiesewetter, München
- Isabel Kiesewetter, München
- Claudia Kiessling, Witten
- Christin Kleinsorgen, Hannover
- Harald Klüter, Mannheim
- Holger Knoth, Dresden
- Ralf Kohal, Freiburg i. Br.
- Sarah König, Würzburg
- Eduard Kraft, München
- Klaus-Dietrich Kröncke, Düsseldorf
- Carsten Kruschinski, Hannover
- Susanne Kühl, Ulm
- Thomas Kühnlein, Erlangen
- Sandy Kujumdshiev, Leipzig
- Felicitas Lahner, Bern (CH)
- Wolf Langewitz, Basel (CH)
- Lorenz Lehmann, Heidelberg
- Ronny Lehmann, Heidelberg
- Holger Lenz, München
- Teresa Loda, Tübingen
- Sabine Ludwig, Berlin
- Björn Lütcke, Erlangen
- Jörg Marienhagen, Regensburg
- Maren März, Leipzig
- Richard März, Wien (AT)
- Jan Matthes, Köln
- Benjamin Mayer, Ulm
- Andreas Möltner, Heidelberg
- Sören Moritz, Köln
- Brigitte Müller-Hilke, Rostock
- Sabine Nabecker, Bern (CH)
- Zineb Miriam Nouns, Berlin
- Frank Nürnberger, Frankfurt/Main
- Falk R. Ochsendorf, Frankfurt/Main
- Wolfgang Öchsner, Ulm
- Wolfgang Paik, München
- Dorothea Penders, Berlin
- Ricardo Patricio Peréz Anderson, München
- Harm Peters, Berlin
- Swetlana Philipp, Jena
- Anysia Poncelet, Heidelberg
- Wolfgang Prodinger, Wien (AT)
- Sabine Ramspott, München
- Gresa Ranxha, Hannover
- Eva Rasky, Graz (AT)
- Markus Rentsch, München
- Matthias Richter, Halle/Saale
- Jörg Roche, München
- Katrin Rockenbauch, Leipzig
- Marco Roos, Erlangen
- Thomas Rotthoff, Düsseldorf
- Caroline Ruiner, Trier
- Stefanie Sasaki-Sellmer, Magdeburg
- Thomas C. Sauter, Bern (CH)
- Thorsten Schäfer, Bochum
- Stefan Schauber, Oslo (N)
- Christian Scheffer, Witten
- Claudia Schlegel, Bern (CH)
- Carolin Schmid, Heidelberg
- Anita Schmidt, Erlangen
- Tabea Schmidt-Ott, London (GB)
- Martina Schmitz, Münster
- Kai Schnabel, Bern (CH)
- Teresa Schreckenbach, Frankfurt/Main
- Sebastian Schubert, Neuruppin
- Nikolai Schuelper, Göttingen
- Henrike Schulze, Hannover
- Katrin Schüttpelz-Brauns, Mannheim
- Thomas Shiozawa, Tübingen
- Anne Simmenroth, Würzburg
- Angelika Simonsohn, München
- Marc Sohrmann, Lausanne (CH)
- Andreas Sönnichsen, Wien (AT)
- Cord Spreckelsen, Aachen
- Sven Staender, Zürich (CH)
- Daniel Steinmann, Freiburg i. Br.
- SylvèreStörmann, München
- Christoph Stosch, Köln
- Ulrich Stößel, Freiburg i. Br.
- Sylvia Stracke, Greifswald
- Irmgard Streitlein-Böhme, Freiburg i. Br.
- Fabian Stroben, Berlin
- David Taylor, Liverpool (GB)
- Valentin Terhoeven, Heidelberg
- Anna-Lena Thies, Münster
- Nils Thiessen, Köln
- Stefan Titz, Heidelberg
- Marie-Laurance Tremblay, Maastricht (NL)
- Gert Ulrich, Zürich (CH)
- Elisa Vietz, München
- Caroline Wachtler, Stockholm (S)
- Ursula Walkenhorst, Osnabrück
- Marc Weidenbusch, München
- Martin Weigl, München
- Mark Weinert, München
- Birgit Wershofen, München
- Jürgen Westermann, Lübeck
- Simone Weyers, Düsseldorf
- Stefanie Wiemer, Leipzig
- Andreas Winkelmann, Neuruppin
- Stefan Wirth, München
- Ulrich Woermann, Bern (CH)
- Alexandra Zech, München
- Danmei Zhang, München
- Stine Ziegler, Hamburg
- Cornelia Ziegler, Hannover
- Hanna Zimmermann, München
- Stefan Zimmermann, Hamburg
- Jan Zottmann, München
- Michaela Zupanic, Witten
- Tycho Zuzak, Herdecke

